# Past, present, and future of non-pharmacological interventions for autism spectrum disorder: A global bibliometric and thematic analysis (2001–2025)

**DOI:** 10.1097/MD.0000000000051164

**Published:** 2026-10-16

**Authors:** Xiaowei Feng, Yu Song, Xuecheng Li, Yibei Yang, Dong Zhang, Yanfeng Zhang, Shanguang Zhao

**Affiliations:** a School of Football, Hainan Normal University, Haikou, Hainan, China; b School of Physical Education and Sports, Central China Normal University, Wuhan, Hubei, China; c School of Physical Education & Sports Science, South China Normal University, Guangzhou, Guangdong, China; d Adapted Physical Activity + Laboratory, South China Normal University, Guangzhou, Guangdong, China; e School of Physical Education, Hainan Normal University, Haikou, Hainan, China; f College of Physical Education, Quanzhou Normal University, Quanzhou, Fujian, China; g National Physical Fitness and Scientific Fitness Research Center, Beijing, China; h Department of Physical Education, Shanghai Maritime University, Shanghai, China.

**Keywords:** autism spectrum disorder, bibliometric analysis, developmental support, early intervention, evidence-based practice, family involvement, non-pharmacological interventions, thematic evolution

## Abstract

**Background:**

Non-pharmacological interventions (NPIs) have become increasingly important in addressing the complex and heterogeneous needs of individuals with autism spectrum disorder (ASD), particularly in light of the limitations associated with pharmacological treatments.

**Objective:**

This study aimed to systematically map the global research landscape and thematic evolution of NPIs for ASD from 2001 to 2025 using bibliometric approaches.

**Methods:**

A total of 341 English-language articles were retrieved from the Web of Science Core Collection. Microsoft Excel and Biblioshiny were employed to perform descriptive analysis, co-authorship network mapping, keyword co-occurrence analysis, and thematic evolution analysis based on Keywords Plus.

**Results:**

The findings indicate a sustained increase in research output, particularly after 2011, with publication volume peaking in 2024 and citation impact continuing to rise. Music therapy, applied behavior analysis, and repetitive transcranial magnetic stimulation emerged as the most influential intervention approaches. The United States led in both publication volume and academic impact, followed by China and the United Kingdom. Thematic evolution analysis revealed a gradual shift from behavior-oriented frameworks toward mechanism-informed and context-sensitive approaches, with increasing attention to early intervention, population heterogeneity, and family involvement.

**Conclusion:**

Research on NPIs for ASD is undergoing a transition toward more integrative, individualized, and evidence-based paradigms. Future studies should prioritize longitudinal designs, cross-cultural validation, and the translation of mechanistic insights into scalable, real-world interventions.

## 1. Introduction

In recent years, autism spectrum disorder (ASD) has become increasingly prevalent worldwide, presenting growing challenges not only for those diagnosed but also for their families and healthcare systems at large.^[1]^ The World Health Organization estimates that roughly 0.76% of children globally are affected by ASD, with a notably higher prevalence observed in high-income countries.^[2]^ In the United States, data from the Centers for Disease Control and Prevention reveal a striking upward trend: the proportion of children identified with ASD has climbed from 1 in 150 in the year 2000^[3]^ to 1 in 31 by 2022.^[4]^ This sharp rise highlights the intensifying public health implications of ASD and the pressing need for effective intervention strategies.

ASD is a neurodevelopmental disorder marked by persistent deficits in social interaction and communication, along with restricted, repetitive behaviors and interests.^[5]^ Individuals with ASD frequently experience comorbid challenges such as mood dysregulation, self-injurious behavior, sleep disturbances, and anxiety.^[6,7]^ Although pharmacological treatments may help manage some of these symptoms – such as anxiety, hyperactivity, and aggressive behavior – their effectiveness is often limited and may come with notable side effects.^[8]^ Therefore, non-pharmacological interventions (NPIs) are increasingly recognized as a major research direction of ASD intervention research, especially in improving social skills, language development, cognitive ability, and daily adaptive behavior.^[9]^

NPIs encompass a broad spectrum of approaches, including natural therapies, complementary and alternative medicine, lifestyle modifications, and psychosocial support strategies.^[10]^ In the context of ASD, commonly studied NPIs include behavioral interventions such as applied behavior analysis (ABA),^[11]^ developmental therapies like developmental, individual differences, relationship-based model/floortime therapy,^[12]^ language and communication interventions,^[13]^ sensory integration therapy,^[14]^ music therapy,^[15]^ exercise intervention,^[16]^ parent-child training,^[17]^ virtual reality assisted therapy,^[18]^ and other forms. Many of these approaches have demonstrated positive effects by enhancing neuroplasticity and behavioral regulation.^[19]^ Particularly in early developmental stages, NPIs are considered critical for fostering gains in language, social engagement, and adaptive skills.^[20]^

With the integration of disciplines such as neuroscience, developmental psychology, rehabilitation science, special education, and artificial intelligence, NPIs are becoming increasingly diversified and refined. This interdisciplinary convergence has contributed to a shift in ASD intervention models – from single-modality treatments to personalized, systematic, and multidimensional strategies.^[21]^

Despite these developments, the field of NPIs for ASD remains broad, heterogeneous, and rapidly evolving. In the past 3 decades, hundreds of studies have been published, and several systematic reviews and meta-analyses have attempted to summarize this field, providing valuable insights for evaluating the intervention effect and optimizing the practice path (e.g.,^[8,22–25]^). However, these studies mostly focus on specific types of NPIs (such as ABA, music therapy, or exercise intervention) or specific groups (such as preschool children or low-functioning individuals), and lack systematic quantitative analysis of the global development trend, knowledge structure, research hotspots, and evolution paths of the whole research field.

Unlike traditional reviews that rely on manual selection and subjective synthesis, bibliometric analysis offers an objective, quantitative approach to mapping the structure and evolution of scientific knowledge.^[26,27]^ By analyzing large volumes of academic publications, bibliometrics can reveal patterns in authorship, institutional collaboration, keyword co-occurrence, citation networks, and thematic evolution, providing a macro-level understanding of a field’s intellectual development.^[28]^ Bibliometric studies have already been applied to various topics in ASD research, including inflammation,^[29]^ genetic risk factors,^[30]^ intestinal flora,^[31]^ executive function of ASD,^[32]^ and so on. However, there is currently a lack of comprehensive bibliometric analysis specifically focused on NPIs for ASD.

Against this backdrop, the present study aims to map the bibliometric landscape and evolutionary trajectory of NPIs for ASD from 2001 to 2025, using data retrieved from the Web of Science. Specifically, the study seeks to:

1. Identify the most productive and influential authors, countries, institutions, and journals;
2. Mine core research topics and emerging trends through keyword co-occurrence and cluster analysis;
3. Track the temporal evolution of this field to reveal the potential future development direction.

Through this comprehensive analysis, we aim to understand the development process and future development direction of NPIs in ASD from a macro level, to provide a reference for future research, practice, and policy formulation.

## 2. Methods

### 2.1. Data sources and retrieval strategies

The bibliometric data of this study are taken from the Web of Science Core Collection (WoSCC), which is one of the most authoritative and widely used academic databases for bibliometric analysis.^[33]^

The 1st search was conducted on March 1, 2026, and the last search was conducted on May 4, 2026, to minimize potential bias caused by database updates. Considering that title-field searches (TI) can reduce false-positive results and keep unrelated articles to a tolerable minimum,^[34]^ we adopted a title-field retrieval strategy combining topic-related keywords to comprehensively identify publications related to NPIs in ASD. The following Boolean operators were applied:

TI = (“autism*” or “autism spectrum disorder*” or “ASD*” or “autistic disorder*” or “autistic spectrum disorder*”) AND (“music therapy” or “play therapy” or “applied behavior analysis” or “TEACCH Training” or “relationship development intervention” or “manual therapy” or “massage” or “acupuncture” or “Yoga” or “exercise intervention therapy” or “structured game on expressing language” or “transcranial magnetic stimulation” or TMS or “hyperbaric oxygen therapy”).

A total of 518 records were initially retrieved. In accordance with the preferred reporting items for systematic reviews and meta-analyses guidelines,^[35]^ a series of exclusion criteria were subsequently applied to ensure the originality, coherence, and relevance of the final dataset (Fig. 1).

**Figure 1.**
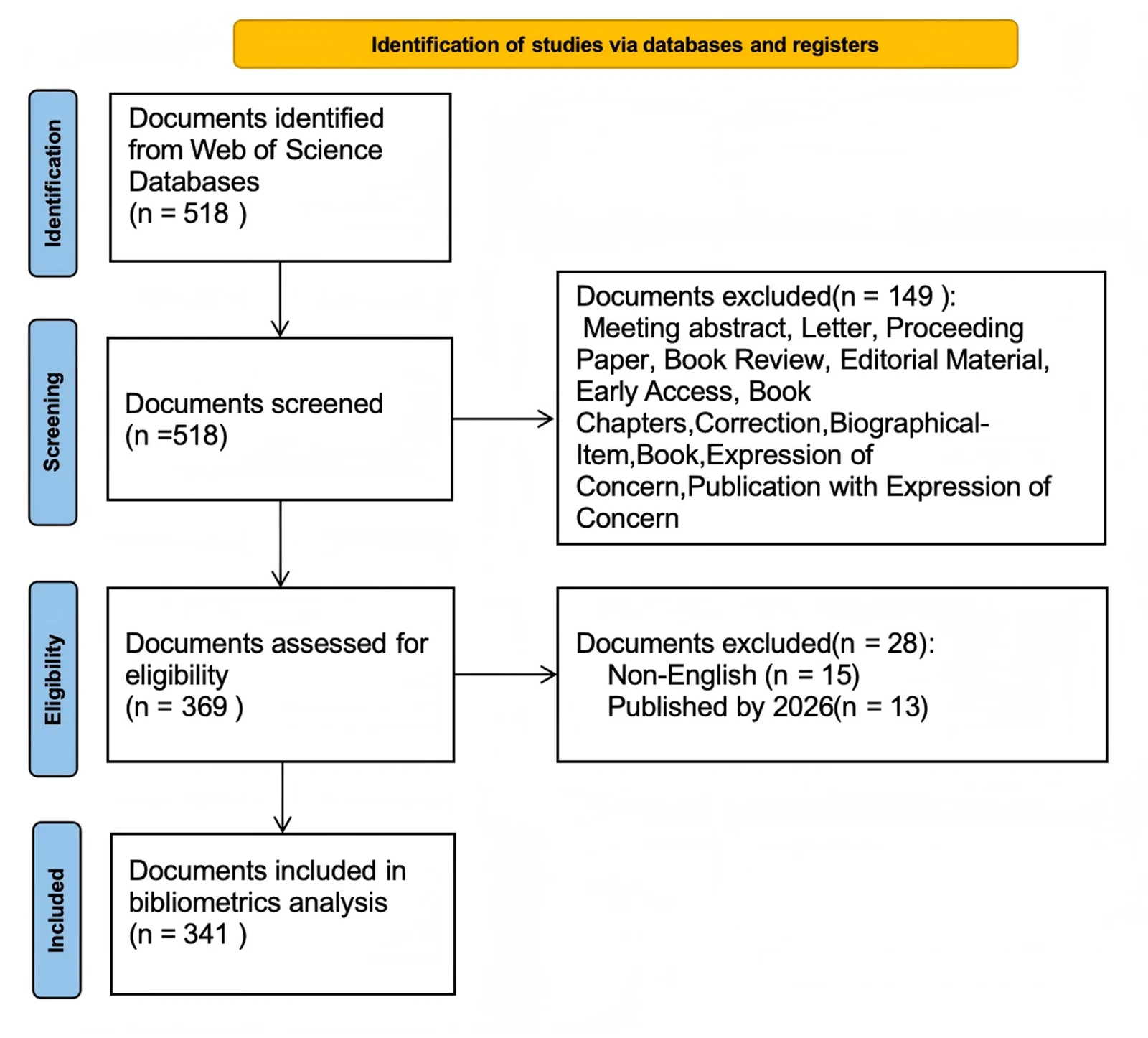
The PRISMA flowchart of the literature screening process. PRISMA = preferred reporting items for systematic reviews and meta-analyses.

First, 149 records were excluded based on document type, including meeting abstracts, proceedings papers, editorial materials, letters, book chapters, early access articles, and other non-article document types. This resulted in 369 eligible records.

Subsequently, 13 articles published in 2026 were removed to avoid potential bias associated with incomplete or continuously updated data. In addition, 15 non-English publications were excluded to ensure consistency and comparability, as English-language studies are commonly adopted as the standard in bibliometric analyses.

After applying all inclusion and exclusion criteria, a total of 341 articles were retained for the final bibliometric analysis, comprising 269 original research articles and 72 review articles. Original studies were defined as empirical investigations presenting novel findings, whereas review articles were characterized by the synthesis and critical evaluation of existing literature.

### 2.2. Bibliometric tools and analytical techniques

To ensure a comprehensive and systematic analysis, we employed the following tools and techniques:

1. Microsoft Excel 2019: It was used to organize and summarize descriptive data obtained from the WoSCC, including information on authors, countries, institutions, and journals. These data facilitated the examination of publication trends and patterns of international collaboration within the field of NPIs in ASD. Information regarding journal impact factor (IF), journal citation reports (JCR) categories, and category quartiles was obtained from the *JCR Science Edition 2024*, which provides standardized metrics for evaluating the quality and influence of scholarly journals.
2. Datawrapper (https://www.datawrapper.de/tables, visited on May 4, 2026): It is used to generate a global map of publication distribution. This visualization illustrates the geographic contributions of various countries and regions to the body of research on NPIs for ASD.
3. Biblioshiny (based on the Bibliometrix R package, University of Naples Federico II): It was utilized to conduct advanced bibliometric analyses. Specifically, it was employed to apply Bradford law to identify core journals, map national collaboration networks to assess the intensity of international research partnerships, and visualize thematic evolution over time. Through high-frequency keywords and theme trend analysis, Biblioshiny helped reveal shifts in research focus and emerging themes in the field of NPIs for ASD.

### 2.3. Ethical considerations

Because this study is based on the secondary analysis of published literature and does not involve human subjects or confidential data, it does not need the approval of the institutional review board or an informed consent procedure.

## 3. Results

### 3.1. Publishing trend analysis

A total of 341 articles published between 2001 and 2025 were included for analysis, based on the predefined search strategy and eligibility criteria. Collectively, these publications accrued 7780 citations, yielding an average of 22.82 citations per article (CPA) and an overall H-index of 48. As shown in Figure 2, the period from 2011 to 2025 contributed 307 articles, accounting for approximately 90% of the total output. The number of publications peaked in 2024 (n = 38), while citation counts continued to rise and reached their highest level in 2025 (n = 1138). Overall, the consistent upward trajectory in both productivity and citation impact reflects the growing scholarly attention and increasing maturity of this research domain.

**Figure 2.**
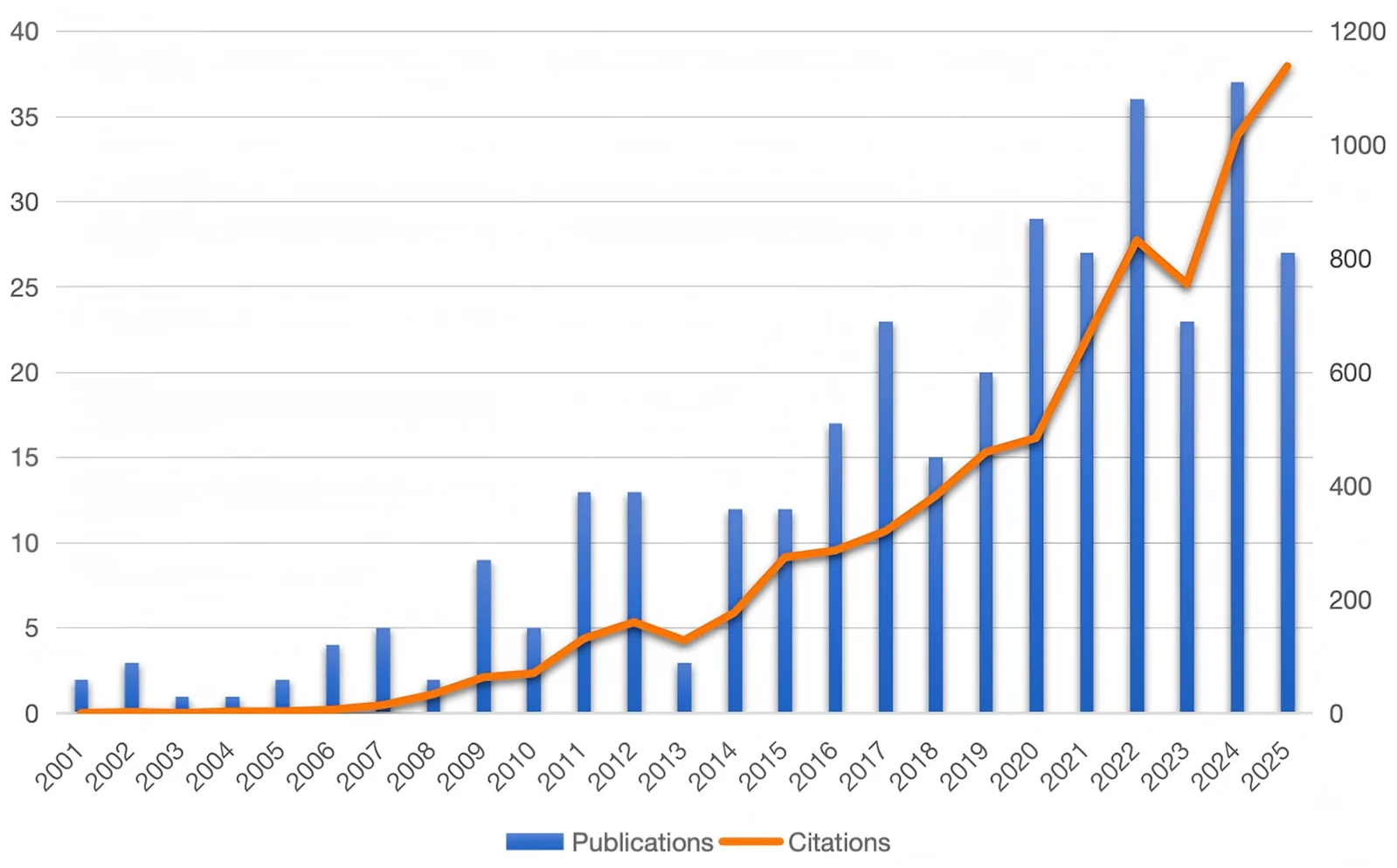
Research publications and citations trends of NPIs on ASD. ASD = autism spectrum disorder, NPIs = non-pharmacological interventions.

### 3.2. Literature source analysis

In bibliometric research, Bradford law describes the centralized distribution of scientific literature, whereby a small number of core journals publish the majority of high-impact research, while a larger number of peripheral journals contain only a few relevant articles.^[36]^ Consistent with this pattern, the literature on NPIs in ASD spans 181 scientific journals, with a subset of 13 core journals emerging as the most prolific sources (Fig. 3). Although these core journals represent only 7.18% (13/181) of the total number of sources, they collectively contributed 115 articles, accounting for 33.72% (115/341) of the total included literature. This concentration underscores the central role of a few high-yield journals in disseminating influential research in this field.

**Figure 3.**
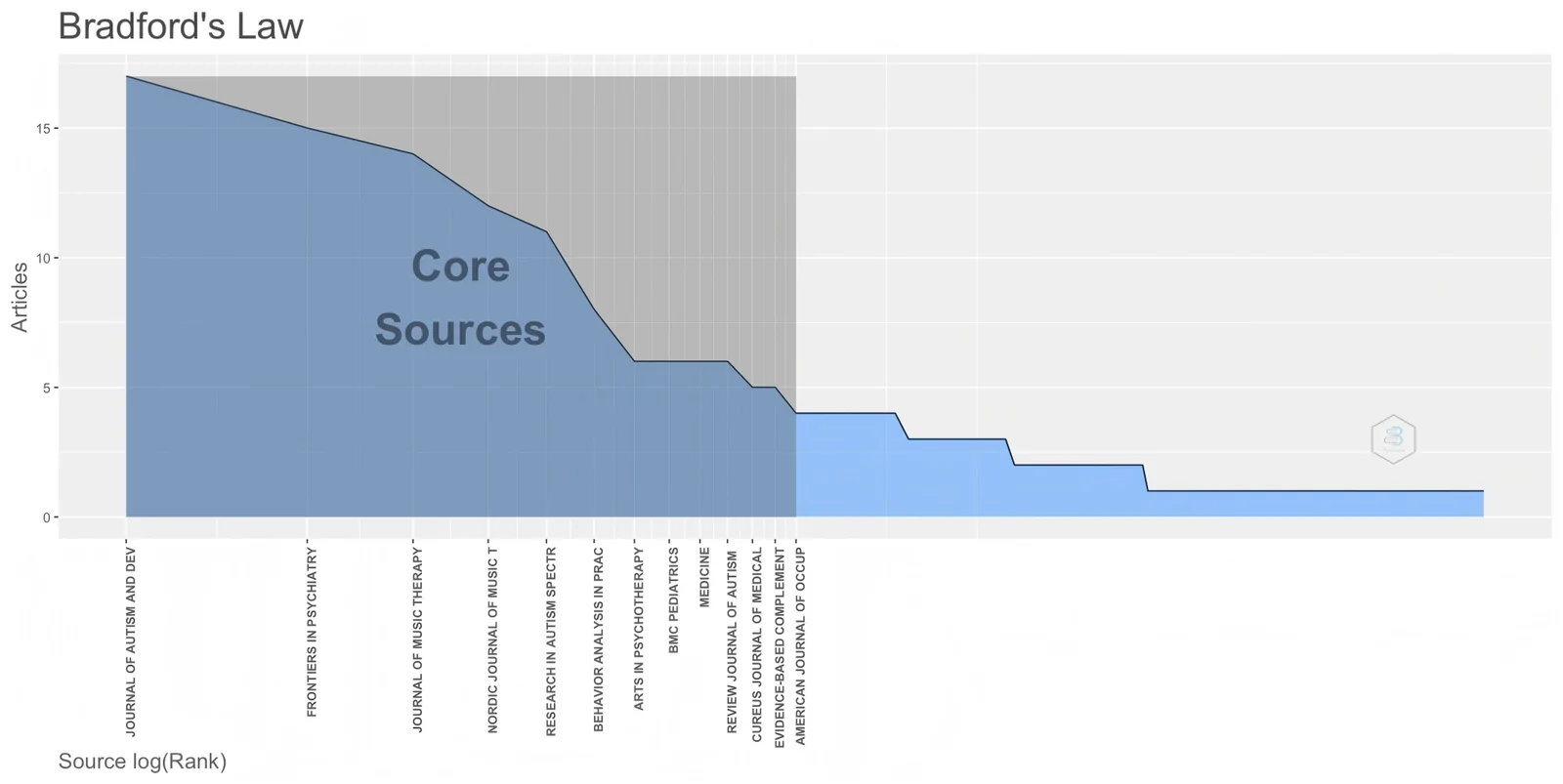
The core journals of NPIs on ASD are based on Bradford law. ASD = autism spectrum disorder, NPIs = non-pharmacological interventions.

To further examine the characteristics of these core journals, we collected relevant metrics from the *Journal Citation Reports (JCR) Science Edition 2024* (Table 1). In terms of category quartile, the 13 core journals are distributed as follows: 1 in Q1, 8 in Q2, 3 in Q3, and 1 in Q4. Among them, the *Journal of Autism and Developmental Disorders* published the highest number of articles (n = 17), accounting for 4.985% (17/341) of the total included literature. *Frontiers in Psychiatry* reported the highest IF among these journals, with an IF of 3.2.

**Table 1:** The top 13 productive journals of NPIs on ASD.

Journals	Articles (%)	IF 2024	CQ 2024	JCR category
Journal of Autism and Developmental Disorders	17 (4.985)	2.8	*Q*2	Psychology, developmental
Frontiers in Psychiatry	15 (4.399)	3.2	*Q*2	Psychiatry
Journal of Music Therapy	14 (4.106)	0.8	*Q*4	Rehabilitation
Nordic Journal of Music Therapy	12 (3.519)	1.2	*Q*3	Rehabilitation
Research in Autism Spectrum Disorders	11 (3.226)	2.1	*Q*1	Education, special; psychiatry; psychology, developmental; rehabilitation
Behavior Analysis in Practice	8 (2.346)	1.7	*Q*3	Psychology, clinical
Arts in Psychotherapy	6 (1.760)	1.5	*Q*2	Psychology, clinical; rehabilitation
BMC Pediatrics	6 (1.760)	2	*Q*2	Pediatrics
Medicine	6 (1.760)	1.4	*Q*2	Medicine, general & internal
Review Journal of Autism and Developmental Disorders	6 (1.760)	3	*Q*2	Psychology, developmental
Cureus Journal of Medical Science	5 (1.466)	1.3	*Q*2	Medicine, general & internal
Evidence Based Complementary and Alternative Medicine	5 (1.466)	2.65	*Q*3	Integrative & complementary medicine
American Journal of Occupational Therapy	4 (1.173)	2.1	*Q*2	Rehabilitation

ASD = autism spectrum disorder, CQ = category quartile, IF = impact factor, JCR = journal citation reports, NPIs = non-pharmacological interventions.

The subject categories of these journals indicate that research on NPIs for ASD is predominantly situated in interdisciplinary domains, including developmental and clinical psychology, psychiatry, rehabilitation medicine, pediatrics, special education, and integrative and alternative medicine. This distribution highlights the highly multidisciplinary nature of the field, reflecting the complex, cross-cutting approaches required to address the diverse needs of individuals with ASD.

### 3.3. Analysis of highly cited documents

As shown in Table 2, the top 10 most highly cited publications in the field of NPIs for ASD primarily focus on 3 major types of interventions: music therapy, ABA, and repetitive transcranial magnetic stimulation (rTMS), with music therapy emerging as the most prominent. Most of these studies were published from 2006 to 2014, suggesting that this period marked a particularly active phase of research and scholarly attention.

**Table 2:** The characteristics of the top 10 highly cited documents of NPIs on ASD.

TC	Article titles	Journals	Published years	Countries	IF 2024		CQ 2024
215	Music therapy for people with autism spectrum disorder (Geretsegger et al., 2014)	Cochrane Database of Systematic Reviews	2014	Denmark	9.4		*Q*1
160	Emotional, motivational and interpersonal responsiveness of children with autism in improvisational music therapy (Kim et al., 2009)	Autism	2009	South Korea	5.6		*Q*1
155	Applied behavior analysis treatment of autism: the state of the art (Foxx, 2008)	Child and Adolescent Psychiatric Clinics of North America	2008	USA	2.2		*Q*3
124	A double-blind, randomized trial of deep repetitive transcranial magnetic stimulation (rTMS) for autism spectrum disorder (Enticott et al., 2014)	Brain Stimulation	2014	Australia	8.4		*Q*1
122	Applied behavior analysis as treatment for autism spectrum disorder (Roane et al., 2016)	Journal of Pediatrics	2016	USA	3.5		*Q*1
119	Music therapy for autistic spectrum disorder (Gold et al., 2006)	Cochrane Database of Systematic Reviews	2006	Norway	9.4		*Q*1
117	Effects of improvisational music therapy vs enhanced standard care on symptom severity among children with autism spectrum disorder the TIME-A randomized clinical trial (Bieleninik et al., 2017)	JAMA – Journal of The American Medical Association	2017	Norway	55		*Q*1
113	Effects of low frequency repetitive transcranial magnetic stimulation (rTMS) on gamma frequency oscillations and event-related potentials during processing of illusory figures in autism (Sokhadze et al., 2009)	Journal of Autism and Developmental Disorders	2009	USA	2.8		*Q*2
102	Family-centred music therapy to promote social engagement in young children with severe autism spectrum disorder: a randomized controlled study (Thompson et al., 2014)	Child Care Health and Development	2014	Australia	2	*Q*2	
100	Effects of a music therapy group intervention on enhancing social skills in children with autism (LaGasse, 2014)	Journal of Music Therapy	2014	USA	0.8	*Q*4	

ASD = autism spectrum disorder, CQ = category quartile, IF = impact factor, NPIs = non-pharmacological interventions, TC = total citations.

In terms of geographic contribution, the United States and European countries (particularly Norway and Denmark) contributed the majority of highly cited work, while Australia and South Korea also made notable contributions. This pattern reflects the dominance of developed regions in producing high-impact research in this field. Notably, the majority of highly cited articles appeared in *Q*1 journals with high IF, such as the *Cochrane Database of Systematic Reviews* (IF = 9.4) and *JAMA-Journal of The American Medical Association* (IF = 55). This reflects the substantial influence of rigorous systematic reviews and randomized controlled trials (RCTs) in shaping the field and driving evidence-based practice.

### 3.4. Productivity analysis of countries/regions and institutions

Based on data retrieved from the WoSCC, researchers from 54 countries or regions have contributed to the literature on NPIs for ASD (Fig. 4). The United States leads in publication volume (n = 140), followed by China (n = 65) and the United Kingdom (n = 27).

**Figure 4.**
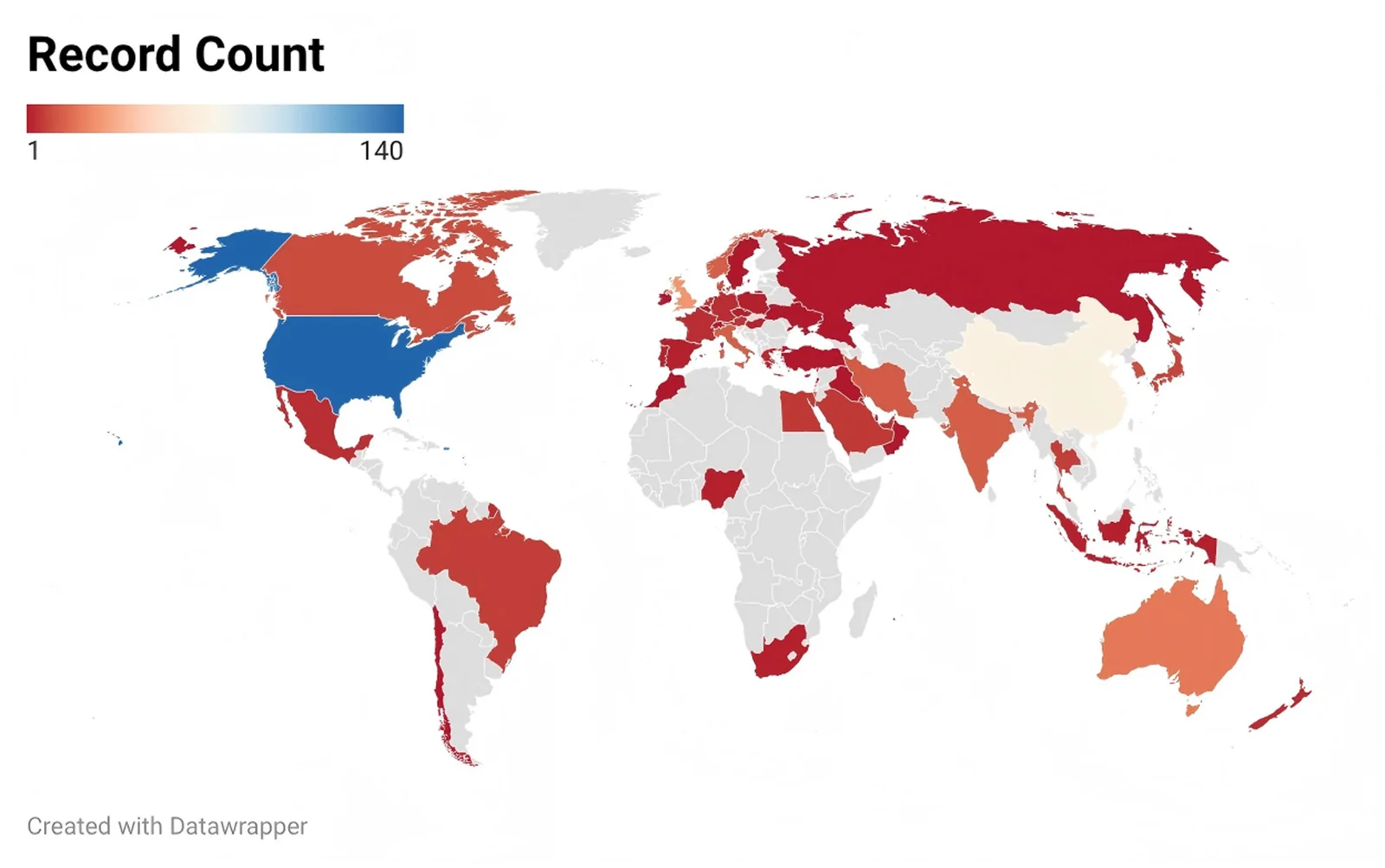
Geographical distribution of research publications on NPIs in ASD. ASD = autism spectrum disorder, NPIs = non-pharmacological interventions.

In addition, the top 12 countries/regions show significant disparities in both geographical distribution and academic output (Table 3). The United States ranks 1st with 140 publications, maintaining a dominant position, with total citations (TC) of 3907 and an H-index of 37, reflecting its leading role in both research productivity and academic influence. At the institutional level, Harvard University and the University of California System each contributed 10 publications, ranking among the most productive institutions.

**Table 3:** Top 12 countries/regions and their leading institutions in research on NPIs for ASD.

Countries	Articles*	TC	H-index	CPA	Top country institutions*	Top institution articles (%)
USA	140	3907	37	27.91	Harvard University University of California System	10 (7.143%)
China	65	786	15	12.09	Beijing Normal University	7 (10.769%)
United Kingdom	27	724	16	26.81	Bangor University University of London	4 (14.815%)
Australia	20	923	15	46.15	Monash University	7 (35.000%)
India	14	154	7	11.00	Svyasa Yoga University	3 (21.429%)
Israel	14	679	11	48.50	University of Haifa	7 (50.000%)
Norway	14	1117	13	79.79	University of Bergen	4 (28.571%)
Italy	14	381	9	27.21	Irccs Fondazione Stella Maris University of Pisa	3 (21.429%)
Iran	13	196	5	15.08	Hamadan University of Medical Sciences Shahid Beheshti University	3 (23.077%)
Canada	10	326	8	32.60	University of Toronto	7 (70.000%)
Denmark	10	740	8	74.00	Aalborg University	9 (90.000%)
South Korea	10	449	8	44.90	Jeonju University	4 (40.000%)

ASD = autism spectrum disorder, CPA = citations per article, NPIs = non-pharmacological interventions, TC = total citations.

* Parallel countries/institutions publish the same number.

China ranks 2nd in publication volume (n = 65); however, its TC (n = 786) and H-index (n = 15) remain substantially lower than those of the United States, indicating that although research activity is increasing, its global academic impact is still developing. Beijing Normal University stands out as a key contributor within China.

Although the publication outputs of the United Kingdom (n = 27) and Australia (n = 20) are lower than those of the United States and China, their citation impact remains relatively high, with CPA values of 26.81 and 46.15, respectively. This suggests that both countries produce research of considerable quality and influence. Bangor University and the University of London in the United Kingdom, as well as Monash University in Australia, serve as major institutional contributors.

Italy (n = 14) and India (n = 14) demonstrate moderate research productivity, although their citation impact differs. Italy shows a relatively balanced performance (TC = 381; CPA = 27.21), whereas India exhibits comparatively lower citation impact (TC = 154; CPA = 11.00), indicating differences in research influence despite similar publication outputs.

Notably, Israel and Norway, each with 14 publications, demonstrate exceptionally high citation impact. Israel achieved a total of 679 citations (CPA = 48.50), while Norway reached 1117 citations with an outstanding CPA of 79.79, highlighting their strong international academic influence. The University of Haifa in Israel and the University of Bergen in Norway each contributed the most national outputs, indicating a high level of institutional concentration.

Other countries, such as Iran, Canada, Denmark, and South Korea, contributed relatively fewer publications. However, in some cases, research output is highly concentrated within leading institutions. For example, the University of Toronto accounted for 70% of Canada’s total output, reflecting a centralized research structure. Similarly, Aalborg University contributed 90% of Denmark’s publications, indicating strong institutional dominance.

Overall, the field demonstrates a global development pattern characterized by the joint participation of North America, Europe, and Asia. High-impact research is predominantly led by institutions in the United States and Europe, while some smaller countries achieve substantial academic influence through high citation impact despite limited publication output.

Figure 5 further shows the network structure of international cooperation, which is divided into 3 clusters:

**Figure 5.**
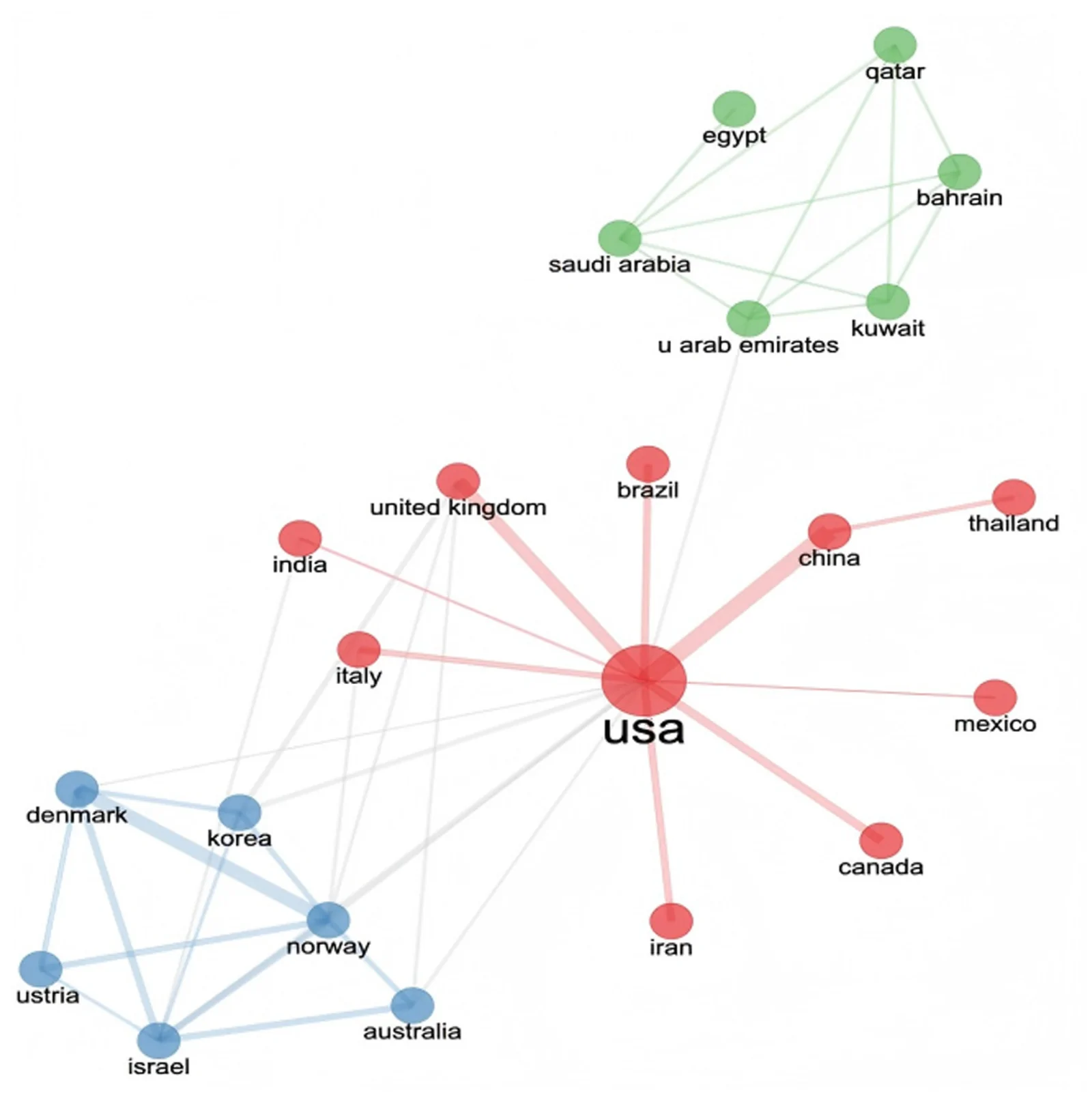
National cooperation network of NPIs research in ASD. ASD = autism spectrum disorder, NPIs = non-pharmacological interventions.

1. Red cluster: It highlights the close cooperation between the United States and China, Canada, Brazil, the United Kingdom, India, Iran, Italy, Mexico, and Thailand.
2. Blue cluster: Including Austria, Norway, Denmark, Australia, Israel, and South Korea.
3. Green cluster: It consists of Egypt, the Bahamas, the United Arab Emirates, Qatar, Kuwait, and Saudi Arabia.

These international collaborations have played a critical role in advancing global research on NPIs for ASD, fostering knowledge exchange, methodological diversity, and cross-cultural innovation.

### 3.5. Analysis of leading authors

In the field of NPIs research on ASD, the distribution of leading authors reveals clear disparities in geographical distribution and academic impact (Table 4). Based on a threshold of at least 5 publications, the top 14 most productive authors are primarily affiliated with institutions in the United States, Canada, Norway, Australia, China, and Israel. Among these, the United States contributes the largest number of leading authors (n = 5), further confirming its dominant position in this research domain.

**Table 4:** Top 14 authors in the NPIs research of ASD.

Authors	Countries	Articles	H-index	TC
Christian Gold	Norway	13	12	1057
Manuel F Casanova	USA	12	11	505
Peter Enticott	Australia	9	9	476
Sokhadze, Estate	USA	9	8	317
Elefant, Cochavit	Israel	7	7	567
Paul B Fitzgerald	Australia	7	7	381
Geretsegger, Monika	Norway	7	7	556
Xiaoli Li	China	6	5	116
Stephanie H. Ameis	Canada	5	4	138
Blumberger, Daniel M	Canada	5	4	168
Paul E Croarkin	USA	5	5	202
Zafiris J. Daskalakis	USA	5	5	188
Desarkar, Pushpal	Canada	5	4	138
Tarbox, Jonathan	USA	5	5	170

ASD = autism spectrum disorder, NPIs = non-pharmacological interventions, TC = total citations.

Norwegian scholar Christian Gold ranks 1st, with 13 publications, an H-index of 12, and a TC of 1057, highlighting his substantial academic influence and sustained contribution to music therapy research. Other highly productive authors, such as Manuel F. Casanova (USA) and Peter Enticott (Australia), have made significant contributions to brain stimulation techniques and neurobiological mechanisms, highlighting the interdisciplinary and methodologically diverse nature of NPIs research.

Although several authors have similar publication outputs (e.g., 6 authors with 5 publications each), their citation impact varies considerably. For instance, Elefant (Israel) and Geretsegger (Norway) demonstrate relatively high citation counts (TC = 567 and 556, respectively), whereas some authors with comparable productivity exhibit lower citation impact. This discrepancy suggests that research influence is not solely determined by publication volume but is also shaped by research focus, methodological rigor, and topic relevance.

Overall, the author distribution reflects a pattern characterized by both concentration and diversity. While a small number of highly influential scholars drive the development of key research themes, a broader group of contributors supports the continued expansion and diversification of the field.

### 3.6. High-frequency keywords and theme evolution analysis

#### 3.6.1. High-frequency keyword analysis

To identify the current research focus within the field of NPIs for ASD, we utilized Biblioshiny to create a Keywords Plus word cloud (Fig. 6). Keywords Plus are terms algorithmically extracted from the titles of cited references rather than the titles of the articles themselves.^[37]^ Compared to author-assigned keywords, these terms often reveal broader and more descriptive thematic trends.^[38]^

**Figure 6.**
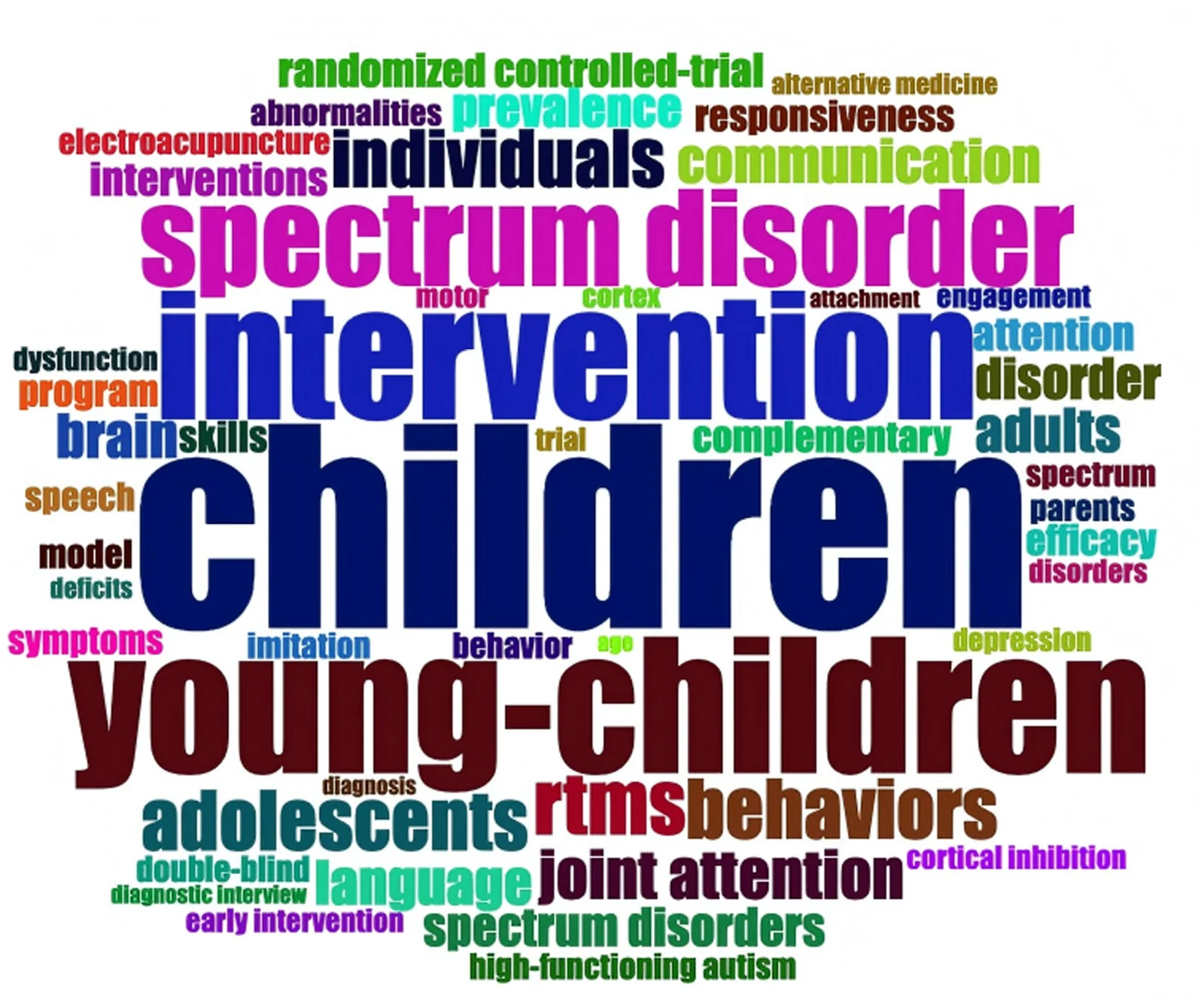
The word cloud of NPIs research of ASD. ASD = autism spectrum disorder, NPIs = non-pharmacological interventions.

The resulting word cloud indicates that intervention strategies targeting children dominate the current research landscape. Terms such as “children” (78 times) and “young-children” (52 times) appear most frequently, underscoring the fact that early childhood remains the primary focus of NPIs’ research in ASD. In contrast, terms like “adolescents” (23 times) and “adults” (17 times) are comparatively infrequent, suggesting that interventions for older age groups remain underexplored and require further attention.

The prominence of terms such as “intervention” (49 times) reflects the field’s conceptual grounding in practical therapeutic applications. Among specific modalities, the frequent appearance of “rTMS” (24 times), “electroacupuncture” (10 times), “complementary” (12 times), and “alternative medicine” (8 times) suggests a growing academic interest in noninvasive neuromodulation and integrative therapeutic approaches. Furthermore, keywords such as “randomized controlled-trial” (13 times) and “double-blind” (10 times) indicate a shift toward more methodologically rigorous and empirically validated research designs, underscoring the field’s progression toward evidence-based practice.

Regarding intervention targets, the frequent recurrence of terms such as “joint attention,” “communication,” “language,” “speech,” “responsiveness,” and “behaviors” demonstrates a continued emphasis on core ASD symptoms, particularly in the domains of social communication and interaction. Simultaneously, the increasing visibility of neuroscience-oriented terms like “brain,” “cortex,” and “cortical inhibition” signals an expanding focus on the neural underpinnings of intervention efficacy.

In summary, current research in NPIs for ASD is characterized by 4 key trends: a strong focus on child populations, diversification of intervention approaches, rising methodological sophistication, and growing attention to underlying neural mechanisms.

#### 3.6.2. Theme evolution trend analysis

To further elucidate the developmental trajectory of NPIs research on ASD, a temporal evolution analysis of Keywords Plus was conducted (Fig. 7). The results reveal a dynamic, stage-wise progression characterized by shifts in population focus, intervention strategies, and conceptual frameworks.

**Figure 7.**
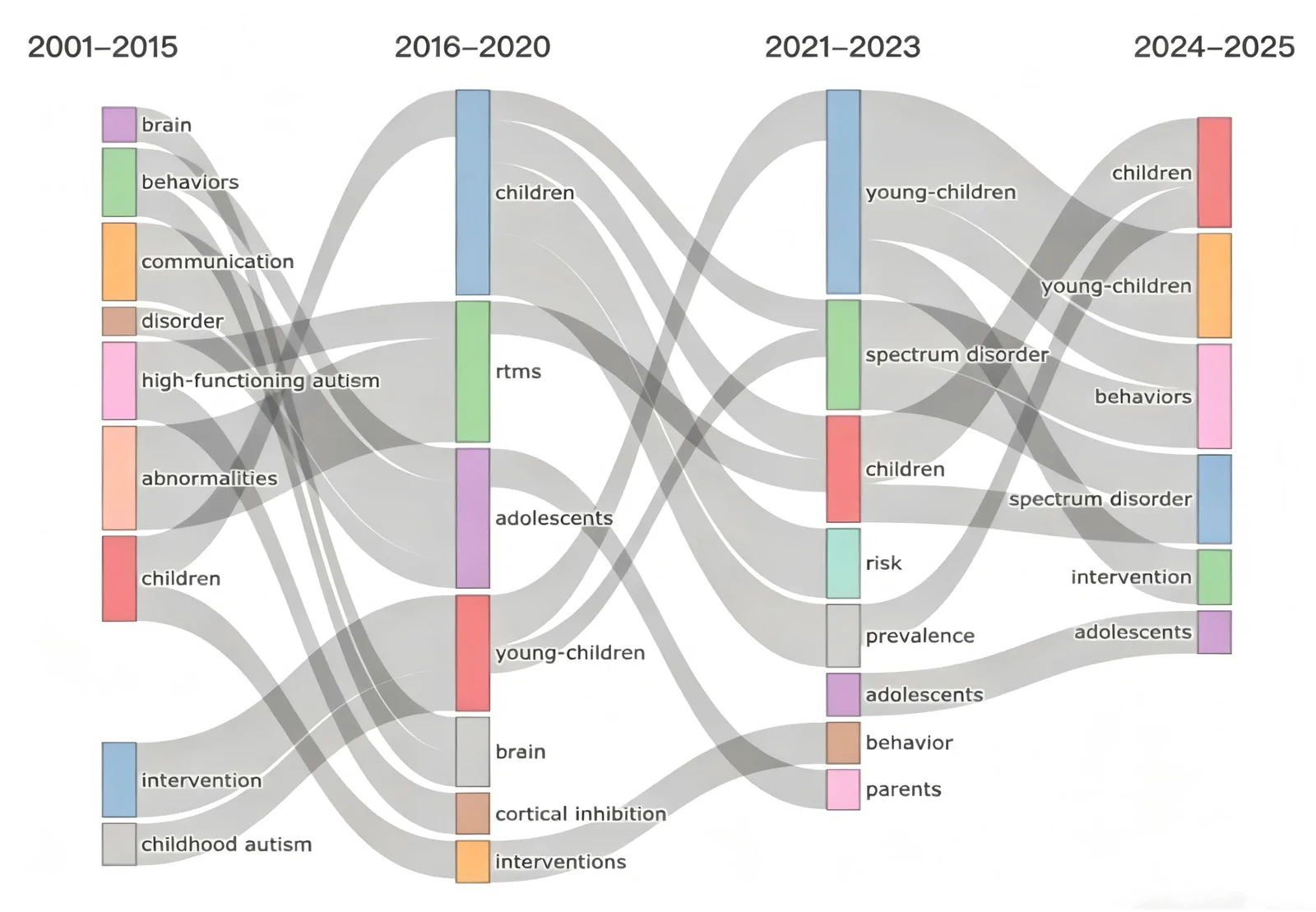
Theme evolution trend diagram of NPIs research in ASD. ASD = autism spectrum disorder, NPIs = non-pharmacological interventions.

##### 3.6.2.1. Phase Ⅰ (2001–2015): Early behavioral focus and foundational constructs

During the initial phase, research primarily focused on core ASD-related behavioral and developmental characteristics. Keywords such as “children,” “behaviors,” “communication,” and “high-functioning autism” indicate that early studies were largely concerned with improving social communication^[39,40]^ and behavioral functioning^[41]^ in children with ASD. The presence of “intervention” further suggests that this period laid the foundation for structured therapeutic approaches (e.g.,^[42–44]^).

At the same time, terms such as “brain” and “abnormalities” imply that researchers had begun to explore the neurobiological characteristics underlying ASD (e.g.,^[45–47]^), although this line of inquiry had not yet become dominant. Therefore, this phase can be understood as a foundational period in which behavioral intervention models were gradually established, while early neurobiological explanations began to emerge.

##### 3.6.2.2. Phase Ⅱ (2016–2020): Expansion of target populations and technological integration

In the 2nd phase, the field expanded in both population coverage and intervention modalities. The prominence of “young-children” and “adolescents” suggests that research attention extended from general child populations to more developmentally specific groups (e.g.,^[48–50]^). This suggests an increasing recognition that intervention outcomes may vary across developmental stages, highlighting the importance of age-sensitive intervention design.

Meanwhile, “rTMS” emerged as a central keyword, marking the increasing incorporation of noninvasive neuromodulation techniques into NPIs research (e.g.,^[51,52]^). This shift reflects a growing need to enhance intervention efficacy and to better understand the neural mechanisms underlying treatment effects. The co-occurrence of “brain” and “cortical inhibition” further indicates that studies during this period began to move beyond behavioral outcomes alone and pay greater attention to neurophysiological mechanisms (e.g.,^[53,54]^). This phase therefore reflects a transition from traditional behavioral intervention toward technology-assisted and mechanism-informed approaches.

##### 3.6.2.3. Phase Ⅲ (2021–2023): Disorder-oriented consolidation and contextual expansion

During 2021 to 2023, the thematic structure became more consolidated around ASD as a spectrum condition. Keywords such as “spectrum disorder,” “children,” and “adolescents” suggest that research increasingly emphasized variability across developmental and functional domains, rather than treating the population as homogeneous (e.g.,^[55–57]^). This reflects a conceptual shift from general intervention models toward more differentiated understandings of individual needs, symptom profiles, and developmental characteristics.

The emergence of “risk,” “prevalence,” and “parents” further indicates that research attention expanded from intervention-focused studies toward a broader framework incorporating epidemiological, familial, and population-level perspectives (e.g.,^[58–60]^). This also reflects a growing emphasis on translating epidemiological insights into targeted intervention strategies for heterogeneous ASD populations. In this sense, the field began to integrate clinical, developmental, and population-level perspectives.

##### 3.6.2.4. Phase Ⅳ (2024–2025): Application-oriented integration and refined intervention strategies

In the most recent phase, keywords such as “children,” “young-children,” “behaviors,” “spectrum disorder,” “intervention,” and “adolescents” indicate that the field has entered a more application-oriented stage. Rather than emphasizing a single intervention technique, current research appears to focus on refining intervention strategies for specific populations and symptom domains.

The continued prominence of child-related terms suggests that early intervention remains the core concern (e.g.,^[61,62]^), while the presence of “adolescents” indicates that older age groups are receiving increasing attention (e.g.,^[63,64]^). At the same time, the recurrence of “behaviors” and “intervention” shows that practical therapeutic outcomes remain central (e.g.,^[65,66]^).

This shift reflects a growing emphasis on translating research findings into real-world practice and delivering more targeted, context-specific interventions. This phase, therefore, reflects a maturation of the field, characterized by the integration of population specificity, behavioral targets, and clinically applicable intervention strategies, with increasing attention to personalized and population-specific intervention approaches.

Overall, the thematic evolution reveals a continuous focus on child populations, accompanied by a shift from early behavioral approaches to technology-driven interventions, and ultimately toward integrated, application-oriented frameworks. This trajectory highlights the field’s progression from exploratory research to a more structured and clinically relevant stage of development.

## 4. Discussion

This study conducts a comprehensive bibliometric analysis of NPIs research in the field of ASD. By systematically studying 341 publications from 2001 to 2025, this analysis provides a detailed overview of the development track, theme evolution, and academic influence of this rapidly developing interdisciplinary field. Through quantitative mapping of publication trends, influential authors, institutions, countries, journals, and thematic hotspots, this study contributes to a macro-level understanding of how NPIs research in ASD has progressed over the past 2 decades and where it may be headed.

### 4.1. Publishing trends and growing academic attention

Analysis of publication and citation trends reveals that since 2011, research on NPIs for ASD has entered a phase of rapid expansion. The number of annual publications reached its peak in 2024, while citation counts continued to increase and achieved their highest level in 2025. This surge reflects not only the intensifying global attention to ASD rehabilitation and support but also the rising significance of NPIs in addressing the complex clinical and public health needs associated with the disorder.^[67]^

Importantly, NPIs are increasingly recognized as valuable tools in promoting health equity, particularly for socially and economically vulnerable populations. Families of individuals with ASD often face substantial psychological and financial burdens related to long-term care and chronic management. As such, ensuring that interventions remain affordable, scalable, and accessible is crucial for reducing disparities in care.^[68]^

The accelerated development of this field may also be associated with the growing standardization of evidence-based methodologies and the adoption of emerging technologies in intervention research. Noninvasive neuromodulation techniques such as rTMS have been increasingly explored to modulate core ASD symptoms, with a growing body of studies examining their safety, efficacy, and clinical applicability.^[69]^ In parallel, the integration of wearable physiological monitoring devices into intervention programs has been reported in recent studies, contributing to improvements in personalization, objectivity, and ecological validity in outcome assessments.^[70]^

The integration of these technical approaches not only enriches the forms of intervention but also provides important support for the theoretical advancement and methodological refinement of NPIs research in ASD.

### 4.2. The distribution of core journals reflects strong multidisciplinary integration

The analysis of literature sources indicates that research on NPIs for ASD demonstrates a clear pattern of interdisciplinary integration. The core journals are primarily distributed across fields such as developmental and clinical psychology, psychiatry, rehabilitation medicine, pediatrics, special education, and integrative and complementary medicine. This disciplinary distribution reflects the inherently multifaceted nature of NPIs, which require the integration of clinical, behavioral, and educational perspectives to address the diverse needs of individuals with ASD.

From the distribution of journals, although the number of core journals is relatively limited, they accounted for 33.72% of the total publications, which is consistent with Bradford law, suggesting that a small number of core journals contribute a large proportion of the total literature output.^[36]^ This centralized distribution trend strengthens the aggregation of knowledge in the field and enhances the academic influence of representative journals. This concentration may facilitate knowledge accumulation and improve the efficiency of information dissemination within the field.

Among these, journals such as *Journal of Autism and Developmental Disorders* and *Frontiers in Psychiatry* have emerged as prominent publication venues, reflecting their importance in disseminating research findings related to NPIs for ASD. Their consistent contribution to the literature suggests that they serve as key platforms for scholarly communication and knowledge exchange in this domain. As the field continues to evolve, these journals are likely to play an important role in facilitating interdisciplinary collaboration and supporting the development of more integrated interventions.

### 4.3. Highly cited documents highlight the centrality of mainstream intervention models and rigorous methodologies

The analysis of highly cited literature underscores the academic and clinical prominence of 3 core intervention models in the field of NPIs for ASD: music therapy, ABA, and rTMS. Among these, music therapy appears most frequently, indicating its sustained influence within the field. Notably, most of the highly cited studies were published between 2006 and 2014, suggesting that this period represents a foundational stage during which key intervention models were established. This suggests that early high-impact studies played a foundational role in shaping subsequent research directions in the field.

Subsequent research has continued to build upon and extend these foundational approaches. Recent studies have further explored the effectiveness and applicability of these interventions in diverse contexts, thereby providing additional empirical support for their clinical applicability, particularly in addressing hallmark ASD symptoms such as social communication deficits and repetitive behaviors.^[71–73]^ This pattern indicates that the core intervention models identified in earlier high-impact studies have maintained their importance in ongoing research rather than being replaced by entirely new paradigms.

A prominent feature of these influential studies is the widespread use of rigorous research designs, including RCTs and systematic reviews. Such methodological approaches contribute to the reliability and reproducibility of findings and enhance their academic impact. Consistent with this, the majority of highly cited articles were published in high-impact, *Q*1 journals, such as *Cochrane Database of Systematic Reviews* and *JAMA – Journal of The American Medical Association –* underscoring the field’s steady shift toward a more standardized, evidence-based research paradigm.^[74]^

In terms of geographic contribution, highly cited publications are predominantly produced by researchers from the United States and European countries, particularly Norway and Denmark, with additional contributions from Australia and South Korea. This distribution suggests that high-impact research in this field has been largely driven by developed regions with strong research infrastructure and established clinical research systems. These studies have contributed to the consolidation of key intervention frameworks and the advancement of evidence-based practices in NPIs for ASD.

### 4.4. The distribution of countries and institutions reveals the global research pattern and international cooperation network

The analysis of the distribution of published countries shows that the United States ranks 1st in the world in terms of both publication volume and academic influence, with its research output and citation performance far exceeding those of other countries, thereby highlighting its leading position in the field of autism research. This leading position may be associated with strong research investment, well-developed research infrastructure, and the advantages of interdisciplinary integration. For example, the National Institutes of Health has continuously provided financial support for ASD-related research through programs such as Autism Centers of Excellence, which may have effectively promoted the transformation from basic research to clinical and translational research.^[75]^ This policy orientation may also have contributed to the development of diversified intervention strategies, such as rTMS, ABA, and music therapy, and may have facilitated the publication of research findings in high-impact international journals.

China ranks 2nd in publication volume; however, its citation impact and H-index remain substantially lower than those of the United States. This discrepancy suggests that while China’s research activity is rapidly increasing, its global academic influence is still developing. In contrast, countries such as the United Kingdom and Australia, despite producing fewer publications, demonstrate relatively high citation impact, indicating strong research quality and influence.

It is worth noting that small countries such as Israel and Norway are less productive than the United States and China in the total number of articles published, but they are excellent in average cited frequency and H-index, which highlights their strategic advantage of “winning by quality.” This pattern may be associated with a higher degree of research focus and institutional concentration. For instance, Israel has established integrated multidisciplinary research frameworks, such as the National Autism Research Center, which may contribute to improving the systematicness and practical relevance of ASD diagnosis, intervention, and follow-up research.^[76]^ Similarly, Norway benefits from large-scale longitudinal studies, such as the Norwegian mother, father and child cohort study, which may provide a solid data foundation for high-quality epidemiological and developmental research and thereby enhance the academic impact of its scientific output.^[77]^

Moreover, the structural analysis of the international collaboration network reveals the formation of several relatively stable cooperative clusters among the United States, Europe, and Asia, highlighting the growing significance of international partnerships in autism research. For instance, the “Voice of Autism Speaks” organization in the United States initiated the global autism public health initiative in 2008, aiming to advance the localization and cross-cultural adaptation of early screening tools and family-based intervention programs in low- and middle-income countries. Through global collaboration networks, this initiative may deliver evidence-based intervention strategies tailored to resource-limited settings.^[78]^ Such transnational cooperation may contribute to expanding the diversity of research samples, improving the external validity of research, and facilitating the validation and dissemination of intervention strategies across different cultural contexts, thereby potentially accelerating the integration and development of global ASD research.

### 4.5. The distribution of leading authors reflects thematic concentration and research influence

The analysis of core authors indicates that the field of NPIs research in ASD is increasingly characterized by the emergence of thematic concentrations, particularly around music therapy, neuromodulation, and ABA. For example, the Norwegian team led by Christian Gold and Monika Geretsegger has played a pivotal role in advancing the evidence base for music therapy.^[79–81]^ In the realm of brain stimulation, Manuel F. Casanova (USA)^[82–84]^ and Peter Enticott (Australia)^[85–87]^ have conducted extensive research on rTMS and its application to ASD-related neural and behavioral dysfunctions. Most of these authors have published continuously in international core journals, which may contribute to advancing theoretical development and practical applications in this field.

Moreover, variations in metrics such as the H-index and total citation count among similarly productive authors further suggest that research influence is not solely determined by publication volume but may also be associated with factors such as the novelty of research questions, the depth of inquiry, and the international visibility and applicability of the findings.^[88]^

### 4.6. Evolution of high-frequency keywords and themes reveals shifts in research hotspots and paradigm development

The evolution of high-frequency keywords reflects not only shifts in research topics but also a gradual transformation in the conceptual frameworks guiding NPIs research in ASD. Rather than representing discrete or isolated thematic changes, these trends point to a progressive reorientation in how interventions are understood, evaluated, and applied.

In the early stage, research was predominantly grounded in behavioral and developmental perspectives, with a strong emphasis on observable symptoms and functional outcomes. Improvements in communication, social interaction, and adaptive functioning were widely regarded as primary indicators of intervention effectiveness, reflecting the prevailing influence of behaviorally oriented models. During this period, foundational intervention approaches, including naturalistic developmental behavioral interventions, were further established and empirically validated through a series of RCTs and applied studies (e.g.,^[89–91]^). Despite these advances, the overall research paradigm remained largely outcome-driven, with relatively limited attention to the underlying mechanisms through which interventions exert their effects.

As the field developed, a gradual shift toward mechanism-informed research became evident. The increasing incorporation of neurophysiological concepts and technologies suggests that researchers began to move beyond purely behavioral descriptions toward exploring the biological substrates of intervention effects. This transition may reflect both advances in neuroscience methodologies and a growing recognition that behavioral outcomes alone are insufficient to fully explain intervention efficacy. For instance, the introduction of noninvasive neuromodulation techniques, such as rTMS, was supported by the development of broader clinical frameworks and accumulating empirical evidence.^[92,93]^ Consequently, intervention research began to integrate behavioral and neurobiological perspectives, marking an important step toward a more comprehensive understanding of ASD.

More recently, the field has shown a clear movement toward integrative and context-sensitive frameworks. The growing attention to factors such as population heterogeneity, developmental stages, and family involvement indicates that NPIs research is increasingly informed by ecological and lifespan perspectives.^[94]^ This shift suggests that interventions are no longer conceptualized as standardized protocols applied uniformly across individuals, but rather as adaptive processes that must be tailored to diverse developmental profiles and real-world contexts. In parallel, advances in neuroimaging, eye-tracking, and electrophysiological techniques have opened new avenues for exploring potential biomarkers associated with intervention response, further supporting the move toward more individualized and mechanism-informed approaches.^[95–97]^

At the same time, the current emphasis on practical implementation reflects a broader transition from experimental validation to applied optimization. Rather than focusing primarily on demonstrating whether an intervention works, recent research appears to prioritize how, for whom, and under what conditions interventions can be most effective. The integration of digital technologies, virtual reality, and remote intervention platforms has further enhanced the feasibility of delivering personalized and scalable interventions in real-world settings.^[98,99]^ In addition, increasing attention to family-mediated and ecologically grounded interventions reflects the growing importance of ecological validity and sustainability in intervention design.^[100,101]^

Despite these advances, bibliometric patterns suggest that several emerging NPIs approaches – such as animal-assisted therapy, exercise-based interventions, and other emerging integrative approaches – have not yet formed stable research clusters. Although these approaches have demonstrated promising effects in improving social functioning and emotional regulation,^[102,103]^ they remain relatively fragmented and have yet to achieve consistent theoretical integration. This pattern may be associated with ongoing challenges related to methodological heterogeneity and the limited consolidation of interdisciplinary evidence.

Overall, the thematic evolution of NPIs research in ASD suggests a paradigm shift from behavior-centered models to mechanism-informed approaches, and ultimately toward integrated, application-oriented frameworks. This trajectory reflects the maturation of the field, characterized by increasing theoretical depth, methodological diversity, and practical relevance. Future research may benefit from further integrating multi-level evidence and strengthening the connection between mechanistic understanding and real-world intervention implementation, thereby advancing the development of more precise and context-sensitive intervention strategies.

## 5. Limitations

Although this study provides a comprehensive bibliometric overview of NPIs research in ASD, there are still some limitations.

First of all, the analysis is limited to the articles included in the core collection of Web of Science, which may exclude related research published in other databases (such as Scopus and PubMed) or regional journals.

Secondly, this study only includes English publications, which may ignore the valuable insights of non-English research, especially in countries where NPI practice is increasing.

Thirdly, although bibliometric methods can reveal macro patterns, they cannot evaluate the clinical efficacy or methodological quality of individual studies. Finally, although the theme evolution analysis is rich in information, it is easily influenced by keyword selection and may not be able to fully capture the nuances of concept development in this field.

## 6. Conclusion

This bibliometric and thematic evolution analysis provides a structured synthesis of the development of research on NPIs for ASD over the past 2 decades. The findings reveal sustained growth in publication output, accompanied by increasing thematic diversification and international engagement. Rather than a simple expansion in volume, the field appears to have undergone a gradual conceptual shift – from predominantly behavior-oriented frameworks toward more integrative approaches that incorporate neurobiological mechanisms, individualized intervention strategies, and contextual considerations.

The results further indicate that the knowledge structure of NPIs research in ASD is inherently interdisciplinary, drawing on contributions from psychology, neuroscience, rehabilitation science, and special education. Emerging themes highlight growing attention to early intervention, parent-mediated approaches, and the application of digital technologies, reflecting a broader movement toward more flexible and scalable intervention models. At the same time, the analysis points to several areas that remain underdeveloped, including cross-cultural validation, long-term effectiveness, and the translation of research findings into ecologically valid settings.

Future research may benefit from strengthening longitudinal designs, enhancing the integration of multi-level evidence (e.g., behavioral, neurophysiological, and contextual data), and developing culturally responsive intervention frameworks. In particular, advancing the linkage between mechanistic insights and real-world implementation will be essential for improving both the precision and applicability of NPIs. By mapping the evolution of research themes and identifying emerging directions, this study contributes to a more nuanced understanding of the field and provides a foundation for guiding future research and practice.

## Author contributions

**Conceptualization:** Xiaowei Feng, Shanguang Zhao.

**Data curation:** Xiaowei Feng.

**Formal analysis:** Xiaowei Feng.

**Methodology:** Xiaowei Feng, Shanguang Zhao.

**Writing – original draft:** Xiaowei Feng, Shanguang Zhao.

**Writing – review & editing:** Xiaowei Feng, Yu Song, Xuecheng Li, Yibei Yang, Dong Zhang, Yanfeng Zhang, Shanguang Zhao.
